# *In situ* targeted MRI detection of *Helicobacter pylori* with stable magnetic graphitic nanocapsules

**DOI:** 10.1038/ncomms15653

**Published:** 2017-06-15

**Authors:** Yunjie Li, Xiaoxiao Hu, Ding Ding, Yuxiu Zou, Yiting Xu, Xuewei Wang, Yin Zhang, Long Chen, Zhuo Chen, Weihong Tan

**Affiliations:** 1 Molecular Science and Biomedicine Laboratory, State Key Laboratory of Chemo/Bio-Sensing and Chemometrics, College of Chemistry and Chemical Engineering, College of Biology, Hunan University, Changsha 410082, China; 2 Faculty of Science and Technology, University of Macau, Av. da Universidade, Taipa 999078, Macau; 3 Department of Chemistry and Department of Physiology and Functional Genomics, Center for Research at Bio/nano Interface, Shands Cancer Center, UF Genetics Institute and McKnight Brain Institute, University of Florida, Gainesville, Florida 32611-7200, USA

## Abstract

*Helicobacter pylori* infection is implicated in the aetiology of many diseases. Despite numerous studies, a painless, fast and direct method for the *in situ* detection of *H. pylori* remains a challenge, mainly due to the strong acidic/enzymatic environment of the gastric mucosa. Herein, we report the use of stable magnetic graphitic nanocapsules (MGNs), for *in situ* targeted magnetic resonance imaging (MRI) detection of *H. pylori*. Several layers of graphene as the shell effectively protect the magnetic core from corrosion while retaining the superior contrast effect for MRI in the gastric environment. Boronic-polyethylene glycol molecules were synthesized and modified on the MGN surface for targeted MRI detection. In a mouse model of *H. pylori*-induced infection, *H. pylori* was specifically detected through both T_2_-weighted MR imaging and Raman gastric mucosa imaging using functionalized MGNs. These results indicated that enhancement of MRI using MGNs may be a promising diagnostic and bioimaging platform for very harsh conditions.

## Abstract (web summary)

The effective detection of *Helicobacter pylori* has been a challenge clinically. Here, the authors demonstrate the potential of magnetic nanoparticles for non-invasive diagnosis of *H. pylori* using magnetic resonance imaging *in vivo*.

Gastric cancer is one of the most common malignant diseases, with high incidence in comparison to various tumours^1^. *Helicobacter pylori* infection plays an important role in the development of gastric cancer^2^; accordingly, rapid, painless and accurate detection of *H. pylori* represents an indispensable diagnostic tool. Biopsy-based testing is commonly performed to detect early *H. pylori* infection^3^,^4^. This panel of tests includes culture^5^, urease test^6^, histology^7^ and PCR^8^, all of which provide direct and accurate information. However, biopsy is an invasive and tedious procedure, requiring endoscopy that is prone to missing small lesions and making the patient uncomfortable. Noninvasive tests have also been developed, such as the urea breath test (UBT)^9^,^10^, IgG serology^11^, immunoblot^12^ and antigen stool detection^13^. UBT, based on the activity of urease of *H. pylori*, is the most sensitive and noninvasive test among these traditional methods. Although this method is sensitive and relatively safe, the introduced ^14^C isotope cannot be eliminated from the body, which is harmful. UBT is also an indirect measurement, thus leading to potential false positive results. Meanwhile, several chemical approaches have been devised to target and capture *H. pylori*, including boronic acid derivatives^14^, that bind to peptidoglycan on the cell wall of bacteria. However, the strong acidity inside the epigastric environment still poses many challenges for the accurate and noninvasive detection of *H. pylori in situ*.

Based on its depth of penetration, noninvasiveness and high spatial and temporal resolution, magnetic resonance imaging (MRI) has played an important role in molecular imaging and clinical diagnosis^15^,^16^,^17^,^18^. MRI can give detailed information about abnormal anatomy in a three-dimensional tomographic and real-time manner with high spatial resolution and soft tissue contrast^19^,^20^,^21^,^22^. MRI is normally applied with contrast agents to enhance the signal. Some contrast agents have been widely used for the early detection of disease, including Gd^3+^, Mn^2+^, superparamagnetic iron oxide nanoparticles (SPIONs) and other magnetic nanoparticles^23^,^24^,^25^,^26^,^27^,^28^,^29^,^30^,^31^,^32^. Contrast agent-based MRI together with the targeted molecular moisture could be a promising platform for the noninvasive detection of *H. pylori in situ*. In these magnetic nanomaterials, Gd^3+^ complexes, such as Gd-tetraazacyclododecane-tetraacetic acid (Gd-DOTA), have had wide clinical use, but free Gd^3+^ can accumulate in the body, and Gd-DOTA used at ultralow pH values for detection has rarely been reported^33^. The Mn^2+^-based contrast agent features facile degradation from MnO_2_ and has therefore been proposed as an alternative to overcome the toxicity of Gd complexes^34^. However, the Mn^2+^ ligand cannot effectively protect the Mn^2+^ core in the ultralow pH of the gastric environment, and the further modification of Mn^2+^ is difficult. To achieve a stable contrast effect in an acidic environment, SPIONs usually need to be coated with silicon dioxide^35^, noble metal^36^ or a carbohydrate^37^ that can affect the role of the magnetic core in the proton relaxation of the surroundings or the surrounding water, an important factor of relaxivity^38^. Magnetic graphitic nanocapsules (MGNs) with single-layer or few-layer carbon atoms with a honeycomb lattice^39^,^40^,^41^ only slightly affected the magnetic core to promote the proton relaxation of the surroundings and exhibited excellent robustness under extreme pH and high salt conditions^42^,^43^,^44^. Such stable core–shell structures could be a good choice as a contrast agent for epigastrium MRI imaging and *H. pylori* detection.

Therefore, in this study, we utilized stable MGNs for the first time for the targeted MRI detection of *H. pylori* in the gastric environment. The MGNs demonstrated superior stability for MR imaging in the acidic gastric environment, both *in vitro* and *in vivo*, when compared with conventional SPIONs. To realize targeted *H. pylori* detection, boronic-polyethylene glycol (B-PEG) molecules, which can reversibly bind peptidoglycan from the bacterial cell wall, were synthesized and modified on the MGN surface through simple but strong hydrophobic interactions. In a mouse model of *H. pylori*-induced infection, *H. pylori* was specifically detected with the functionalized MGNs through both T_2_-weighted MR imaging and the Raman imaging of gastric tissue, indicating that the enhancement of MRI using MGNs may be a promising diagnostic and bioimaging platform for very harsh conditions.

## Results

### Synthesis and characterization of MGNs

**Figure 1:**
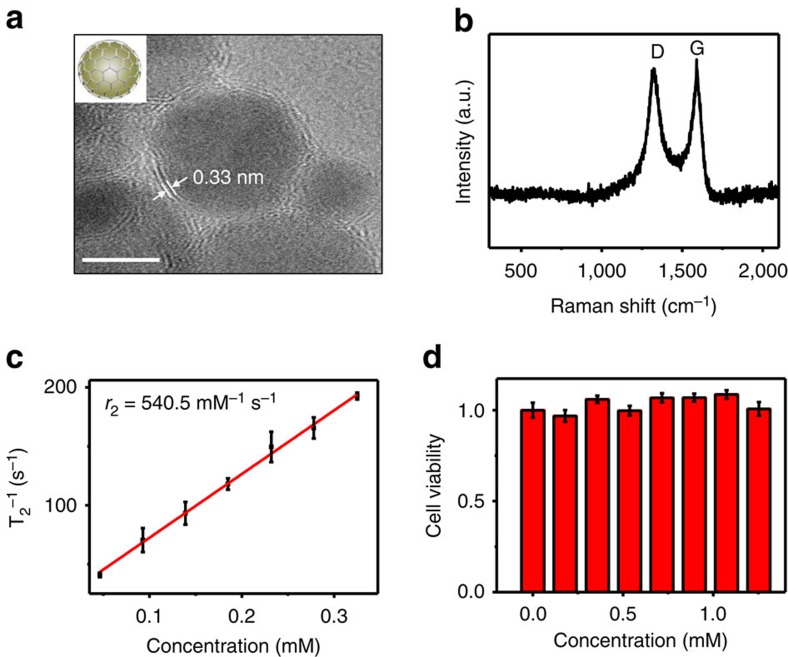
MGN characterization. (**a**) High-resolution TEM (HR-TEM) images of ∼7 nm MGNs. Scale bar, 5 nm. (**b**) Raman spectrum of MGNs (excitation 633 nm), showing the D-band and G-band of graphitic carbon. (**c**) MR T_2_ relaxivity characterization. The *r*_2_ relaxivity value was obtained from the slope of the linear fit (red solid line) of the experimental T_2_ data. (**d**) Cell viability test with MTS assay. Error bars indicate the s.d., *n*=3.

Core–shell MGNs were synthesized through a simple one-pot method. Transmission electron microscopy (TEM) showed that the as-prepared products consisted of uniform nanoparticles with a magnetic core and a multilayered graphitic shell (Fig. 1a). Ultraviolet–visible spectra of the MGNs indicated that the absorbance band located at ∼258 nm originated from the graphitic shell (Supplementary Fig. 1a). The average diameter of the MGNs was ∼7 nm, and high-resolution TEM showed that the outside shell was ∼0.7 nm. The space between the outside shell layers of the MGNs was ∼0.33 nm, consistent with the interlayer distance of graphite and suggested that the magnetic core was encapsulated by few-layer graphene. The hydrodynamic diameter of the MGNs is ∼30 nm (Supplementary Fig. 1b). The MGNs exhibited a nearly neutral charge after treatment with a nitric acid solution to remove the MGNs that were not well isolated by the graphene (Supplementary Fig. 1c). In Raman spectroscopy, the MGNs revealed a graphitic carbon (G) peak at ∼1,600 cm^−1^ and a disordered (D) peak at ∼1,320 cm^−1^ (Fig. 1b). We used C_18_-PEG (5,000, molecular weight) to obtain a stable aqueous dispersion of the MGNs and enhanced their biocompatibility through noncovalent interactions between the graphitic shell and the alkyl chains of the PEG molecules. The graphitic shell isolated the FeCo magnetic core very well and exhibited excellent robustness under an extreme pH environment.

For comparison, superparamagnetic iron oxide nanoparticles were prepared by coprecipitating ferric chloride and ferrous sulfate under sodium hydroxide^45^,^46^. Polyacrylic acid was modified on the SPIONs to improve their solubility and biocompatibility. The average diameter of the SPIONs was ∼9 nm (Supplementary Fig. 1d). The hydrodynamic diameter was nearly 32 nm, with nearly neutral charge (Supplementary Fig. 1e,f).

Next, magnetic properties were investigated. The *r*_2_ relaxivity value of MGN is ∼540.5 mM^−1^ s^−1^ (Fig. 1c), and this is higher than that of SPIONs (135.5 mM^−1^ s^−1^, Supplementary Fig. 2a) based on the effective magnetic relaxations of the proton around the MGNs^38^. The signal intensity of T_2_-weighted phantom images clearly decreased with the increase in Fe concentration, also indicating that the MGNs were superior to SPIONs as an MRI contrast enhancer on T_2_-weighted sequences (Supplementary Fig. 2b). The cytotoxicity of the MGNs was then investigated with the MTS assay. As shown in Fig. 1d, the MGNs exhibited good biocompatibility. Negligible inhibition of proliferation was observed in MGC-803 gastric cancer cells stained with MGNs.

### Stability of MGNs *in vitro* and *in vivo*

**Figure 2:**
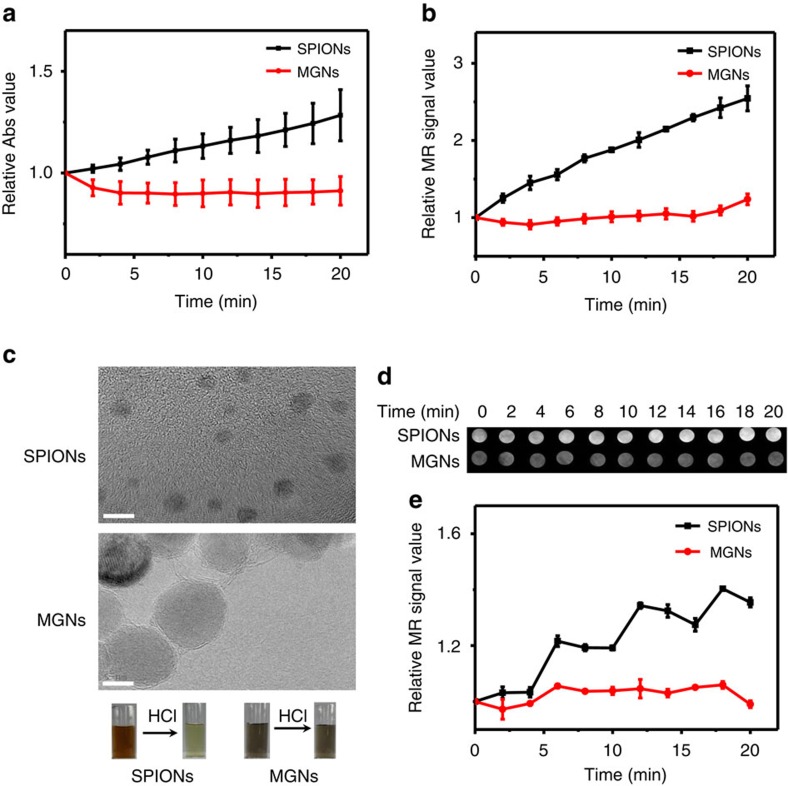
Acid corrosion resistance tests with 1 M HCl. (**a**) The change of ultraviolet–visible absorbance normalized to ∼330 nm of MGNs and SPIONs after the addition of 1 M HCl at 2 min intervals up to 20 min. (**b**) Normalized MR T_2_ relaxivity measurements of MGNs and SPIONs over time. (**c**) Corresponding changes of colour (photos as insert) and high-resolution TEM (HR-TEM) of MGNs and SPIONs after HCl treatment for 20 min. Scale bar, 5 nm. (**d**) T_2_-weighted phantom images of MGNs and SPIONs after the addition of HCl at 2 min intervals up to 20 min. (**e**) Corresponding quantitative data analysis of (**d**) at different times after HCl treatments. Error bars indicate the s.d., *n*=3.

In addition to their high magnetic relaxivity, the chemical inertness of graphene also granted superior stability to the graphene-isolated magnetic nanoparticles. We then assessed the ability of the MGNs to resist acid corrosion. As shown in Fig. 2, the MGNs demonstrated excellent stability in concentrated HCl (1 M) solution, and the robust graphitic shell did not change the solution colour. However, SPIONs were not as stable under identical conditions, and the solution colour shifted from brown to light yellow, which is the colour of Fe^3+^. Figure 2a also displays the clear changes in the ultraviolet–visible absorbance at 330 nm of the SPIONs, while no change was observed for the MGNs. Since SPIONs dissolve, the contrast effect between the magnetic core and water weakened, and the relaxivity decreased (Fig. 2b). T_2_-weighted phantom imaging effects of SPIONs, as shown in Fig. 2c, notably decreased with time, while images of MGNs did not change, even in an acidic environment. We then quantified the T_2_-weighted phantom images, and the stability of MGNs is shown in Fig. 2d. Dynamic light scattering was used to monitor the size and ζ-potential of the SPIONs and MGNs (Supplementary Fig. 3e,f). Based on the production of Fe^3+^, the ζ-potential of the SPIONs shifted from 0.4 to 78 mV. The size of the SPIONs also shifted from 32 to 2 nm, while no such changes were observed in the MGNs, further confirming their superior resistance to acid corrosion. When the concentration of HCl was increased to 4 M, the MGNs demonstrated even better stability than the SPIONs (Supplementary Fig. 3a,d) in such harsh conditions.

**Figure 3:**
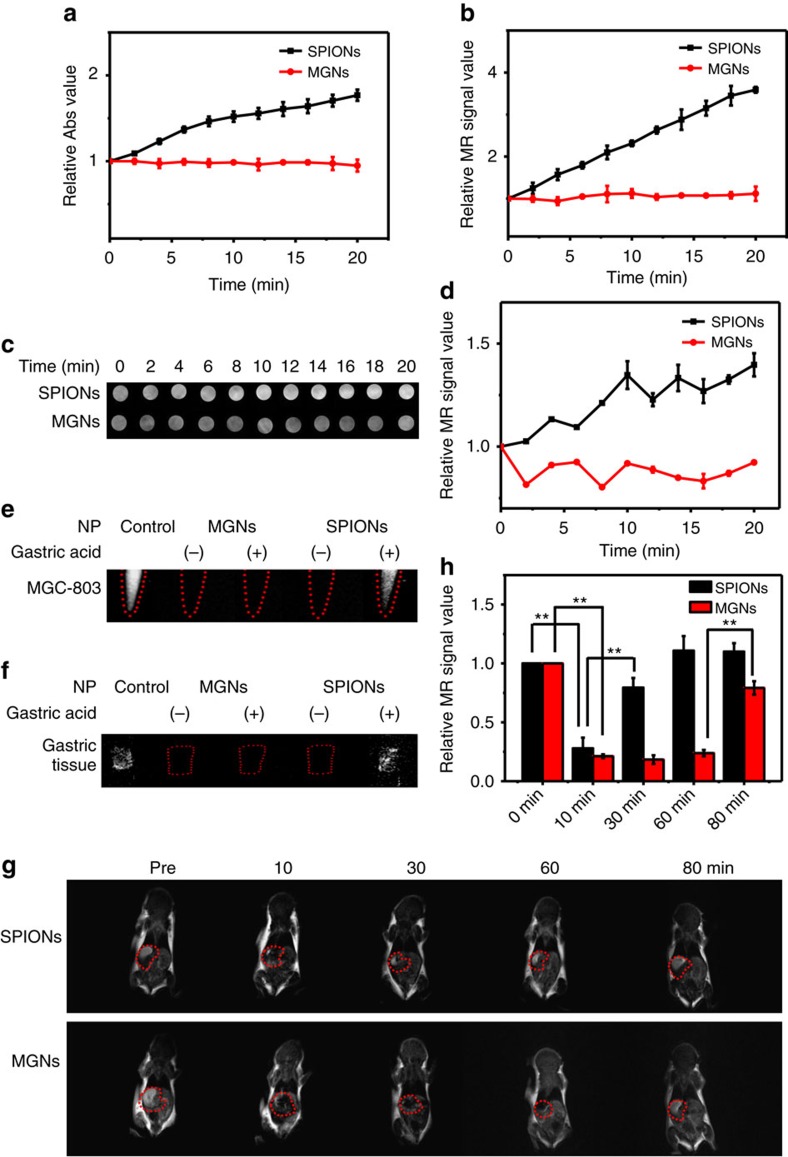
Comparison of stability in the pH=0 gastric acid mimic at different times. (**a**) Ultraviolet–visible absorbance after addition of gastric acid. (**b**) MR T_2_ relaxivity measurements after addition of gastric acid. (**c**) T_2_-weighted phantom images of gastric acid mimic treated with MGNs or SPIONs at different times. (**d**) Corresponding quantitative data analysis of **c**. Error bar indicates the s.d. (**P<0.01 from the two-way analysis of variance (ANOVA) with Tukey’s post-test). T_2_-weighted phantom images of MGNs and SPIONs at different times with and without gastric acid treatment: (**e**) MGC-803 cells, (**f**) tissue of BALB/c mice gastric mucosa, (**g**) BALB/c mice model and (**h**) corresponding quantitative data analysis of mice in **g**. Error bars indicate the s.d., *n*=3.

The real pH value of gastric acid^47^ is ∼0.9–1.5. As a gastric acid mimic, we prepared a pH=0 solution with hydrochloric acid, sodium chloride and pepsin according to USP^48^ to test the stability of the MGNs. Figure 3a,b shows the relative ultraviolet–visible absorbance and T_2_ relaxation time, respectively, of the MGNs and SPIONs under conditions of gastric acid mimicry. T_2_-weighted phantom images of the two nanoparticles in the gastric acid mimic were also recorded (Fig. 3c,d). Much like their performance in pure HCl solution (Fig. 2), the MGNs also demonstrated much better stability than the SPIONs. A pH=1, gastric acid mimic was also prepared, and the results were the same (Supplementary Fig. 4). These results show that the graphitic shell of the MGN had been successfully synthesized, resulting in the protection of the magnetic core against acidic corrosion.

The gastric cancer cell line MGC-803 was also incubated with the MGNs and SPIONs both with and without the gastric acid treatments. As shown in Fig. 3e, without gastric acid treatment, both the MGNs and SPIONs could be used as a T_2_* contrast agent for MR imaging. However, with the gastric acid treatment, only the MGNs retained MRI capability, indicating their potential for application in cancer diagnostics. We also demonstrated the robust MR imaging capability of the MGNs on the gastric tissue samples (Fig. 3f). Again, upon examination of the tissue sections, gastric acid treatment could not destroy the graphitic shell-protected MGNs. We then performed *in vivo* T_2_-weighted imaging by intragastrically inoculating BALB/c mice with MGNs or SPIONs. An external magnet was utilized to prolong the retention of the MGNs and SPIONs in the stomach. As shown in Fig. 3g, SPIONs were rapidly cleared from the stomach, and the contrast effect almost disappeared after 30 min. However, for MGNs, the T_2_-weighted imaging lasted more than an hour and demonstrated superior stability in the mouse stomach. It is also noteworthy that the contrast effect of the MGNs disappeared after 80 min, indicating good biocompatibility. Figure 3h shows the quantification of the *in vivo* MR imaging in Fig. 3g, highlighting the difference between the performances of the MGNs and SPIONs. MGNs demonstrated superior stability *in vitro* and *in vivo*, indicating great potential for MR imaging and detection in the gastric environment.

### Synthesis of B-PEG and *in vitro H. pylori* detection

**Figure 4:**
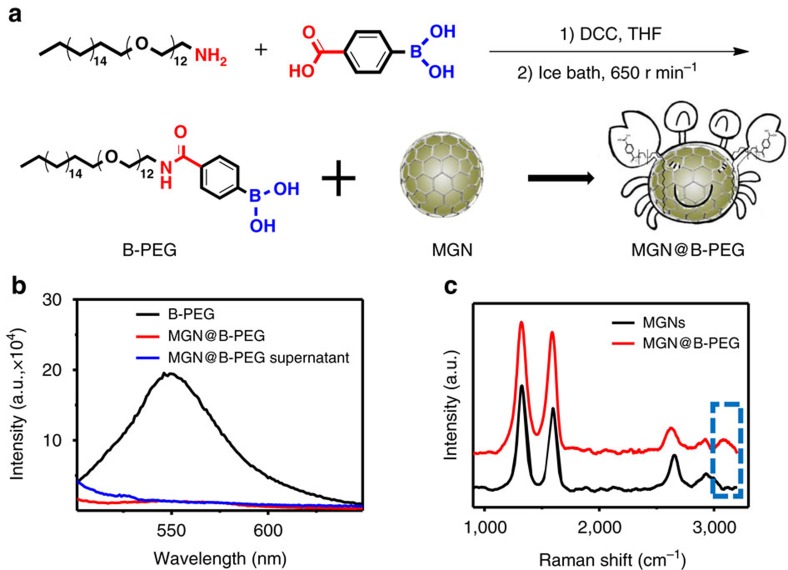
Synthesis and characterization of boronic-polyethylene glycol (B-PEG)-functionalized MGN. (**a**) Schematic illustration of B-PEG synthesis and MGN functionalization. (**b**) Fluorescence characterization of B-PEG-functionalized MGNs. (**c**) Raman spectra of MGN with and without B-PEG functionalization.

*H. pylori* is a Gram-negative bacterium typically found in the stomach. It can cause many diseases such as acute gastritis, peptic ulcers and even gastric cancers^49^. MGNs were utilized for the painless, fast and *in situ* detection of *H. pylori* in the stomach. Boronic acid is a molecule that can reversibly bind peptidoglycan from the cell wall of bacteria, leading to the specific capture of *H. pylori*^14^,^50^,^51^. We synthesized a B-PEG and functionalized it on the surface of MGNs to realize the targeted detection of *H. pylori* in the stomach. The boronic molecule was efficiently synthesized and conjugated on the PEG chain, as indicated in Fig. 4a. Such B-PEG molecules also have a hydrophobic alkyl tail, establishing conditions for their facile functionalization on the surface of MGNs through hydrophobic interactions for *H. pylori-*specific targeting. The synthesized B-PEG was characterized by electrospray ionization (ESI), ^1^H-NMR, ^13^C-NMR and Fourier transform infrared (FTIR) spectroscopy (Supplementary Fig. 5). The peak of 982.9 in ESI demonstrated the generation of B-PEG. The peaks at 4.3 p.p.m. in ^1^H-NMR and 3,215 cm^−1^ in FTIR also demonstrated the formation of an amide bond, indicating the successful synthesis of B-PEG molecules. The graphitic shell of the MGNs could quench the fluorescence of a proximal fluorophore through the fluorescence resonance energy transfer effect^52^. The fluorescence of the boronic acid group on B-PEG substantially decreased after interacting with the MGNs, indicating the successful functionalization of B-PEG on the surface of the MGNs (Fig. 4b). Further Raman characterization exhibited the D- and G-bands of the MGNs and the 3,100 cm^−1^ peak of the OH group^53^ on B-PEG, also indicating the successful functionalization of MGNs with B-PEG molecules.

**Figure 5:**
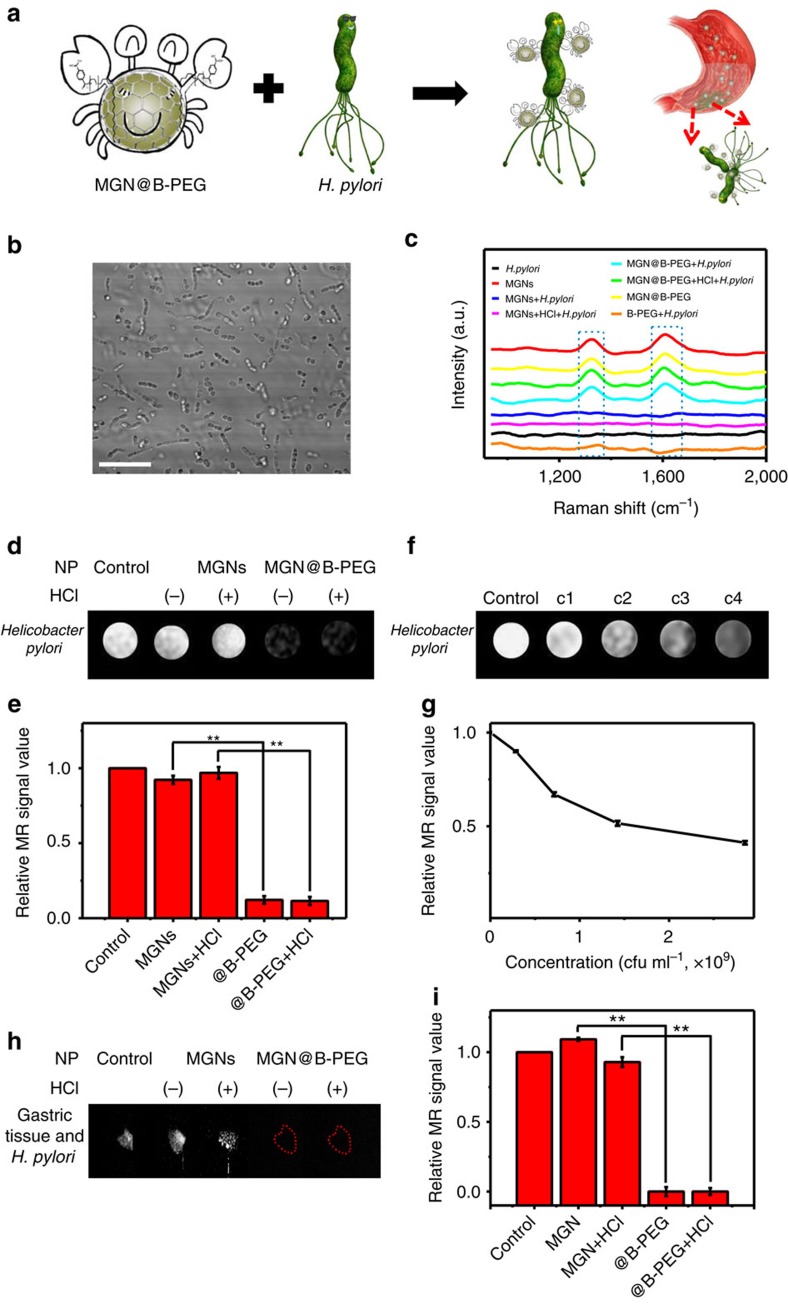
Targeted *in vitro* detection of *H. pylori* with MGN@B-PEG. (**a**) Schematic illustration of *H. pylori* detection with MGN@B-PEG. (**b**) Confocal laser scanning microscopy (CLSM) images of *H. pylori*. Scale bar, 50 μm. (**c**) Raman spectroscopic characterization of *H. pylori* detection with MGN@B-PEG *in vitro*. (**d**) MR images of *H. pylori* with MGN or MGN@B-PEG after different treatments. (**f**) MR images of different concentrations of *H. pylori* with MGN@B-PEG. (**h**) MR images of *H. pylori* colonized on tissue of BALB/c mice gastric mucosa with MGN or MGN@B-PEG after different treatments. (**e**,**g**,**i**) Corresponding data analysis of samples in (**d**,**f**,**h**) with MGN or MGN@B-PEG after different treatments, respectively (**P<0.01 from the two-way analysis of variance (ANOVA) with Tukey’s post-test). Error bars indicate the s.d., *n*=3.

B-PEG-modified MGNs were utilized for *H. pylori* detection. Figure 5a illustrates the strategy for the specific capture of *H. pylori*. B-PEG molecules can reversibly bind peptidoglycan from the bacterial cell wall, much like the pincers of a crab. This allows accelerated aggregation of MGNs, allowing the easy capture of *H. pylori* virions. Figure 5b shows the morphology of *H. pylori* using confocal laser scanning microscopy. The helical structure of *H. pylori* viewed under a microscope resembles lotus roots. We cultured *H. pylori* with or without MGN@B-PEG and then collected the Raman spectra of *H. pylori*. As shown in Fig. 5c, no characteristic peak in the Raman spectra of *H. pylori* was observed when treated with MGN or B-PEG alone. In contrast, when *H. pylori* was treated with MGN@B-PEG, the D-band and G-band of MGNs could be easily detected, demonstrating their superior targeting ability. Furthermore, the characteristic peaks of the graphitic shell were unaffected when MGN@B-PEG was pretreated with HCl, demonstrating the effective capture of *H. pylori* (Fig. 5c). All these results point to MGN@B-PEG having high potential for use as a contrast agent in the gastric environment with superb detection. Figure 5d shows the T_2_-weighted phantom images of *H. pylori* with different treatments. No obvious T_2_* contrast MRI signal was observed with MGNs, while the MR signal darkened when using the MGN@B-PEG as a contrast agent with or without the hydrochloric acid pretreatments. Quantification is shown in Fig. 5e, and MGN@B-PEG demonstrated superior specific detection of *H. pylori*.

By further increasing the concentration of *H. pylori*, more MGNs could aggregate on the surface of *H. pylori*, thereby enhancing the T_2_ imaging signal, as shown in Fig. 5f. The boronic acid group of MGN@B-PEG efficiently aids MGNs in the sensitive capture of *H. pylori* (Fig. 5g). The capture efficiency of the MGN@B-PEG was investigated. Concentrations of *H. pylori* as low as ∼1,000 colony-forming unit (c.f.u.) per ml could be detected with MGN@B-PEG through targeted MRI detection, indicating its superior bacterial-capture efficiency *in vitro* (Supplementary Fig. 6). After the exposure of gastric tissue to *H. pylori*, sections were incubated with MGN or MGN@B-PEG with and without HCl treatment for T_2_-weighted phantom imaging (Fig. 5h). Consistent with the above results obtained in solution, the B-PEG-functionalized MGNs demonstrated excellent *H. pylori* capture efficiency and stability on gastric mucosal tissue (Fig. 5i). Moreover, since the boronic molecule can widely capture bacteria whose cell walls are composed of peptidoglycan, MGN@B-PEG was also utilized to detect *E. coli*. As expected, MGN@B-PEG also demonstrated superior detection efficiency and stability (Supplementary Fig. 7).

### Targeted *in vivo* detection of *H. pylori* with MGNs

**Figure 6:**
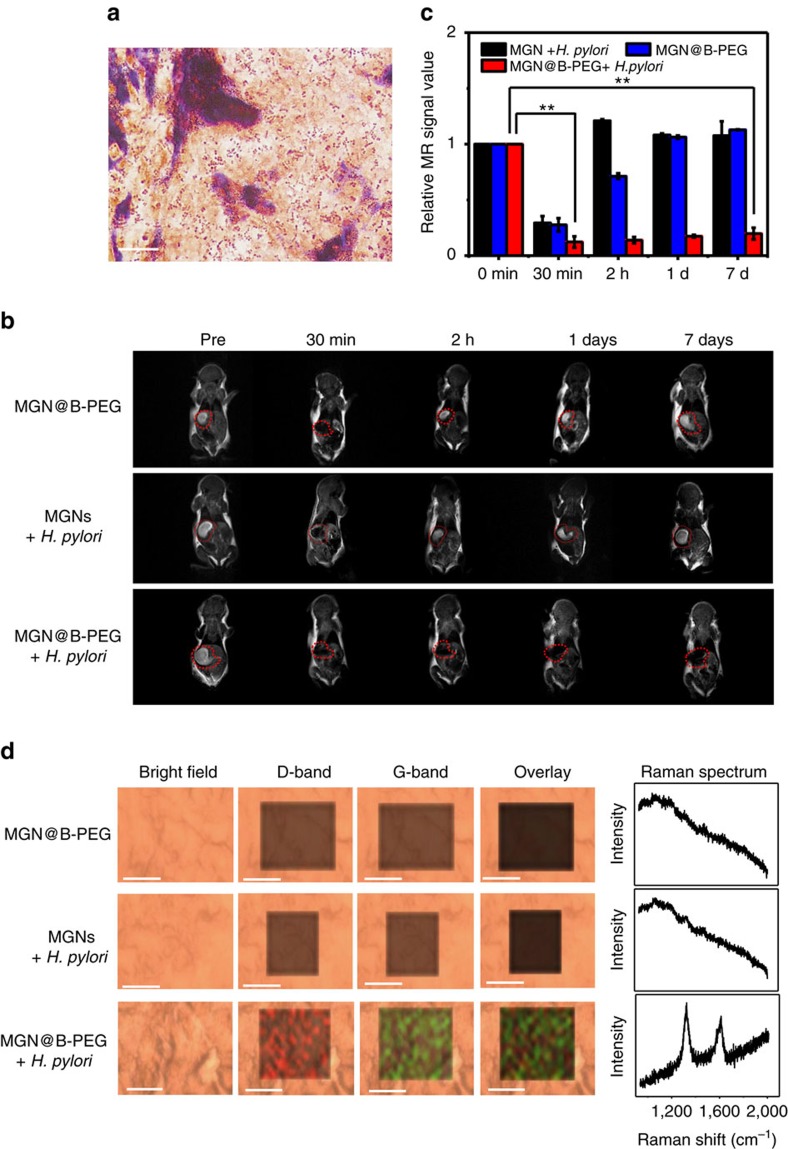
MGN@B-PEG for *H. pylori* detection in infected BALB/c mice. (**a**) Gram staining of a slice from the gastric mucosa of BALC/c mice with *H. pylori* implanted. Scale bar, 10 μm; slice thickness, 10 μm. (**b**) T_2_-weighted images at different times with or without MGN@B-PEG treatments. (**c**) Corresponding data analysis of mice gastric mucosa in (**b**) at different times with or without MGN@B-PEG treatments. Error bars indicate the s.d. (**P<0.01 from the two-way analysis of variance (ANOVA) with Tukey’s post-test). (**d**) Raman images and spectra of mice gastric mucosa used in (**b**). Scale bar, 10 μm; slice thickness, 50 μm. Error bars indicate the s.d., *n*=3.

The capability of the MGNs for *in vivo* targeted detection of *H. pylori* was then explored. BALB/c mice were fasted for 24 h, followed by intragastric administration with 3 × 10^9^ c.f.u. ml^−1^ *H. pylori* for 3 days^54^. The control group was treated with Dulbecco’s phosphate-buffered saline (DPBS). *H. pylori* infection was examined following preparation of stomach tissues in paraffin-embedded sections. With Gram staining, mouse gastric mucosa was stained in red that indicated successful implantation of *H. pylori* in the stomach (Fig. 6a). We then treated the mice with MGNs or MGN@B-PEG by intragastric administration. Without B-PEG functionalization, MGNs were eliminated rapidly after injection, and *H. pylori* could not be detected. However, MGN@B-PEG was found to be a stable contrast agent for T_2_-weighted phantom imaging of gastric mucosa infected with *H. pylori*, and distinct imaging results were stable for 7 days. As demonstrated in Fig. 6b, B-PEG contributed to the capture of *H. pylori* and contributed to the aggregation and retention of MGNs in the stomach for a prolonged period. A noninfected control group was treated with MGN@B-PEG in the same manner as that described above. MRI results showed that MGN@B-PEG could be cleared in <2 h, demonstrating good specificity and low background. Figure 6c shows quantitative analysis of the T_2_ signal from the MRI imaging of the mice stomachs, clearly demonstrating the advantages of utilizing MGN@B-PEG for enhanced T_2_-weighted targeted imaging of *H. pylori in vivo.* In the clinic, there might be different bacterial species that can survive in the stomachs with reduced acid such as in patients with intake of proton-pump inhibitor or with achlorhydria. To identify those bacterial species, the MGNs might need to be modified with other functional molecules such as antibodies, aptamers or other ligands specific to those bacterial species. A comparison for specificity of detection against other bacteria (Supplementary Fig. 8) was investigated with MGNs modified with nucleic acid that can more specifically recognize the *H. pylori*^55^. The MGN–nucleic acid complexes demonstrated superior detection specificity, also indicating that the MGNs are a promising diagnostic and bioimaging platform. To further identify the targeting ability of the MGN@B-PEG, we killed the mice after 7 days and utilized the D-band and G-band of the MGNs for Raman imaging of their gastric tissue (Fig. 6d). The distinct Raman peaks of the graphitic shell of the MGNs could be easily identified in the *H. pylori*-infected gastric mucosa treated with MGN@B-PEG. These results further confirmed the successful targeted detection of *H. pylori in vivo*. The sensitivity of the MGN@B-PEG for *H. pylori* detection *in vivo* was also investigated. Different amounts of *H. pylori* were inoculated into the mice. Through the MGN@B-PEG targeted MRI detection, *H. pylori* levels as low as ∼2,000 c.f.u. ml^−1^ could be detected (Supplementary Fig. 9), demonstrating sensitive bacterial capture *in vivo*. All the *in vivo H. pylori* detections were further examined with Gram staining and urease assay of the gastric mucosa after killing the mice (Supplementary Figs 10–12 and Supplementary Table 1)^56^. The results also confirmed the good sensitivity of the MGN@B-PEG for *in vivo* targeted MRI detection of *H. pylori*.

In conclusion, magnetic graphitic nanocapsules were utilized for the targeted detection of *H. pylori* in the gastric environment. The chemical inertness of the graphene granted superior stability to the graphene-isolated magnetic nanoparticles, even under very harsh acidic/enzymatic conditions of the gastric environment. The magnetic cores of the MGNs were effectively protected from contamination and corrosion by the graphitic shell and maintained enhanced T_2_ contrast imaging efficiency. The MGNs demonstrated superior stability for MR imaging compared to conventional SPION nanoparticles, both *in vitro* and *in vivo*, in the harsh gastric environment. Such stable MGNs was further utilized for the painless, fast and *in situ* detection of *H. pylori* in mouse stomach. For the targeted detection of *H. pylori*, we synthesized a B-PEG molecule with a hydrophobic alkyl tail. This allowed for simple functionalization on the surface of the MGNs through hydrophobic interactions. B-PEG-modified MGNs demonstrated superior targeted detection of *H. pylori* through both enhanced T_2_-weighted MR imaging and Raman imaging of gastric tissue sections. Overall, these acid-resistant, stable MGNs demonstrated superior capability for efficient targeted *H. pylori* detection, showing promise for additional biomedical applications, such as targeted tumour imaging and diagnostics, photothermal therapy and drug delivery in very harsh biological conditions.

## Methods

### Materials and animals

Fumed silica was purchased from Aladdin Industrial Corporation (Shanghai, China). Gastric pepsin was purchased from Sigma-Aldrich China (Shanghai, China). C_18_-PEG_12_-NH_2_ was purchased from Shanghai Zhenzhun Biology Company (Shanghai, China). BALB/c female mice (∼5 to 6 days old) were obtained from the Hunan SLRC Laboratory Animal Co., Ltd (Changsha, China) and used under protocols approved by the Institutional Animal Care and Use Committee of Hunan University. *H. pylori* was donated by Xiangya Hospital (Changsha, China). RPMI-1640 medium was obtained from Thermo Fisher Scientific (Suzhou, China). MTS was purchased from Beyotime Biotechnology Corporation (Shanghai, China). A Gram staining kit was purchased from Qingdao Hi-tech Industrial Park Hope Bio-technology Co., Ltd (Qingdao, China). A mouse urease enzyme-linked immunosorbent assay (ELISA) kit was purchased from Biopeony Beijing Co., Ltd (Beijing, China). Water was double distilled at a resistivity of >18.2 MΩ cm^−1^. Other agents were obtained from Changsha Chemical Reagents Company and were used without further purification.

### Characterization and instruments

TEM images were taken with a JEM-2010 (JEOL, Japan). Ultraviolet–visible spectra were recorded by a UV-2450 spectrophotometer (Shimadzu, Japan). Hydrodynamic diameters and ζ-potentials were measured by a DLS system (Malvern, UK). Cell viability and urease activity assays were measured by a multi-mode microplate reader (Bio-Tek, USA). A Raman imaging microscope system was used to record the Raman spectra with 633 nm laser excitation (Renishaw, UK). A Minispec MQ60 60 MHz TD NMR broadband spectrometer (Bruker, Germany) was used to measure T_2_ relaxivity. T_2_-weighted MR imaging was taken by the 1.5 T small animal MR scanner (Shinning Globe MRI 1.5 T, China). Gram-stained slices were observed using a BX 51 microscope (Olympus, Japan). Confocal laser scanning microscopy was performed using an FV 1000 laser confocal microscope (Olympus, Japan).

### Synthesis of the MGNs and SPIONs

MGN nanoparticles were prepared through a chemical vapour deposition method. The synthesis began by loading Fe(NO)_3_·9H_2_O (0.145 g) and Co(NO)_3_·6H_2_O (0.105 g) onto the fumed silica powder by impregnation in a methanol solution. Silica loaded with metal was dried and placed into a methane chemical vapour deposition chamber for growth. Then, MGNs were synthesized in a methane flow of 150 s.c.c.m. for 5 min at 800 °C. Once cooled to room temperature, we used 10% ethanol and 10% hydrofluoric acid in H_2_O to etch the silica, followed by collecting the MGNs with a magnet.

We prepared the superparamagnetic iron oxide nanoparticles by coprecipitating 1.35 g of ferric chloride and 0.75 g of ferrous sulfate under 1.3 g of sodium hydroxide at 100 °C while stirring for 1.5 h. Then, we modified the SPIONs with polyacrylic acid to increase their biocompatibility.

### T_2_ measurement

We used aqueous solutions of different concentration of MGNs and SPIONs for T_2_ measurement using a 1.5 T MQ 60 scanner at 37 °C. Then, we analysed the data through linear fitting to obtain the relaxivity of the MGNs and SPIONs.

### Changes of ultraviolet–visible spectra and MRI after addition of HCl

To investigate stability in an acid environment, we prepared a 1 M HCl acid solution. We added 400 μl of 1 M HCl into 400 μl of 0.23 mM MGNs (T_2_, 7.2 ms) or 400 μl of 1.01 mM SPIONs (T_2_, 7.2 ms). We performed ultraviolet spectroscopy, T_2_ measurement and MRI phantom imaging every 2 min after the addition of HCl.

### *In vitro* T_2_-weighted imaging

To investigate the stability of the MGNs and SPIONs *in vitro* to enhance the MRI contrast, the MGNs and SPIONs were suspended in HCl solution and incubated for various lengths of time. All the *in vitro* T_2_-weighted magnetic resonance images were acquired using a 1.5 T small animal MRI scanner (at 37 °C) with the following parameters: coil type, spiral coil; Tr, 2 s; Te, 56 ms; the average, 3; field of view, 35 × 45 mm; matrix size, 256 × 256; slice thickness, 1.2 mm; scan duration, 2 min; resolution, 0.16 × 0.16 mm; flip angle, 90°; software for acquisition and analysis and the duration of imaging, shinning MRI.

### MGC-803 cells stained with MGNs and SPIONs

The MGC-803 (Guangzhou Cellcook Biotech Co., Ltd, China), a gastric cancer cell line, was utilized since we focused on the imaging and detection in the stomach. Mycoplasma contamination test was carried out and no contamination was observed. MGC-803 cells were incubated at a density of 2.3 × 10^5^ cells per plate and cultured for 24 h at 37 °C. After 24 h of culture, we incubated the MGC-803 cells with 20 μl of 0.45 mM MGNs (T_2_, 3.9 ms) and 20 μl of 2.5 mM SPIONs (T_2_, 3.9 ms) for 2 h. To detach the cells, 200 μl of trypsin was added to each plate. To each plate, 1 ml of RPMI-1640 culture medium (Gibco) was added. Cells were collected through centrifugation for 3 min at 800 r.p.m. at 4 °C and prepared for T_2_-weighted MR imaging.

### *In vivo* T_2_-weighted imaging

To investigate the MRI contrast in the gastric environment, BALB/c mice ( each group with 3 mice) were anaesthetized using 0.12 ml of chloral hydrate (10 wt%). We treated the BALB/c mice with MGNs (T_2_, 2.5 ms) at a dose of 2.54 mmol per kg and SPIONs (T_2_, 2.5 ms) at a dose of 10.4 mmol per kg of mouse body weight by oral administration, and MRI was performed at the desired time points after injection. A 1.5 T small animal MR scanner (60.875 MHz) was used to monitor the mice for 7 days. All the *in vivo* T_2_-weighted magnetic resonance images were acquired using a 1.5 T small animal MRI scanner (at 37 °C) with the following parameters: coil type, spiral coil; Tr, 500 ms; Te, 19.6 ms; the average, 3; field of view, 35 × 45 mm; matrix size, 256 × 256; slice thickness, 1.2 mm; scan duration, 15 min; resolution, 0.16 × 0.16 mm; flip angle, 90°; software for acquisition and analysis and the duration of imaging, shinning MRI.

### Synthesis of B-PEG

A tetrahydrofuran solution of C_18_-PEG_12_-NH_2_ (159.4 mg, 0.2 mmol) and 4-carboxyphenylboronic acid (43.16 mg, 0.2 mmol) was stirred in an ice bath. The B-PEG product was generated by dropwise addition of one equivalent of *N*,*N*′-dicyclohexylcarbodiimide. After stirring overnight in an ice bath, B-PEG was purified by column chromatography using CH_3_OH and CH_2_Cl_2_ (1:50). The products were then characterized by ESI, NMR and FTIR. For MGN functionalization, 20 μl of B-PEG and C_18_-PEG were added into the solution and ultrasonicated for 1 h. Then, we washed off excess B-PEG and C_18_-PEG, and the product was stored for further use.

### *In vitro H. pylori* detection

We quantified *H. pylori* by ultraviolet–visible spectra, and the concentration of *H. pylori* was ∼2 × 10^9^ c.f.u. ml^−1^ when the absorbance was 1.0 in 600 OD. We then separately incubated *H. pylori* with MGNs and MGN@B-PEG for 30 min at 37 °C in a shaker. We collected *H. pylori* by centrifugation at 2,000 r.p.m. for 10 min. Raman spectroscopy and MRI were used to detect *H. pylori.*

To investigate the limit of detection of the MRI for targeted *H. pylori* detection *in vitro*, we incubated different concentrations of *H. pylori* (C_1_–C_10_: 1,000, 2,000, 5,000, 1 × 10^4^, 5 × 10^4^, 2 × 10^5^, 4 × 10^5^, 5 × 10^5^, 1 × 10^6^, 2 × 10^6^ c.f.u. ml^−1^) with MGN@B-PEG for 30 min at 37 °C in a shaker. Then, T_2_-weighted magnetic resonance images were collected with the MRI scanner.

### *In vivo H. pylori* detection

BALB/c mice were fasted for 24 h, followed by intragastric administration of 3 × 10^9^ c.f.u. ml^−1^ *H. pylori* for 3 days. A control group was treated with DPBS during the same 3 days. We treated BALB/c mice (each group with 3 mice) with MGNs or MGN@B-PEG at a dose of 2 mmol per kg of mouse body weight by oral administration. All BALB/c mice were anaesthetized using 0.12 ml of chloral hydrate (10 wt%) before MR imaging. A 1.5 T small animal MR scanner was utilized to monitor the changes in the stomachs of these mice over 7 days.

To investigate the limit of detection of the MRI for targeted *H. pylori* detection *in vivo*, we inoculated the BALB/c mice with different concentrations of *H. pylori* (C_1_–C_8_: 1,000, 2,000, 5,000, 1 × 10^4^, 5 × 10^4^, 1 × 10^5^, 1.5 × 10^5^, 2 × 10^5^ c.f.u. ml^−1^) for 3 days. Then, we treated BALB/c mice with MGN@B-PEG at a dose of 2 mmol per kg of mouse body weight by oral administration. Before MRI scanning, 0.12 ml of chloral hydrate (10 wt%) was used to anaesthetize the mice. Then, T_2_-weighted magnetic resonance images were collected with the MRI scanner before and 2 days after the MGN@B-PEG treatments.

### Nucleic acid functionalization for specificity of detection

MGNs modified with nucleic acid (MGN-DNA), which can more specifically recognize *H. pylori*, were utilized to investigate the specificity of detection against different bacteria. The DNA sequence is as follows: 5′-AAAAAAAAAGAGACTAAGCCCTCC-3′. The DNA was prepared at 100 μM in 40 μl of DPBS and mixed with 0.018 mmol of MGNs for 30 min at 37 °C. After allowing functionalization, the mixture was exposed to external magnetization for 2 h to collect the MGN-DNA. Then, *Escherichia coli* and *H. pylori* were each incubated with the MGN-DNA solution for 30 min at 37 °C in a shaker. All the bacteria were collected by centrifugation at 2,000 r.p.m. for 10 min. Then, Raman spectroscopy and MRI were used to characterize the bacterial detection.

### Urease comparison tests

Phenol red solution was prepared with 12.0 mg of phenol red in 3 ml of H_2_O. Then, 0.1 M H_2_SO_4_ and 0.1 M NaOH were used to alter the pH of phenol red to 6.6–8.0, and the colour of the solution from yellow to orange. Phenol red solution (30 μl) and H_2_O (800 μl) were added to 200 mg of urea to obtain a yellow solution of urea phenol. Gastric slices from different BALB/c mice were incubated with 10 μl of urea phenol solution. The solution would turn red in the presence of *H. pylori*. A mouse urease ELISA kit was used to detect the urease concentration. Standard or sample solution (50 μl) were added to different wells for urease detection following the ELISA kit instructions. A multimode microplate reader was used to read the absorbance at 450 nm that is related to the concentration of urease.

### Data availability

All the data supporting the findings of this study are available within the article and its Supplementary Information files and from the corresponding author on request.

## Additional information

**How to cite this article:** Li, Y. *et al*. *In situ* targeted MRI detection of *Helicobacter pylori* with stable magnetic graphitic nanocapsules. *Nat. Commun.* **8**, 15653 doi: 10.1038/ncomms15653 (2017).

**Publisher’s note**: Springer Nature remains neutral with regard to jurisdictional claims in published maps and institutional affiliations.

## Supplementary Material

Supplementary Information Supplementary Figures and Supplementary Tables.
