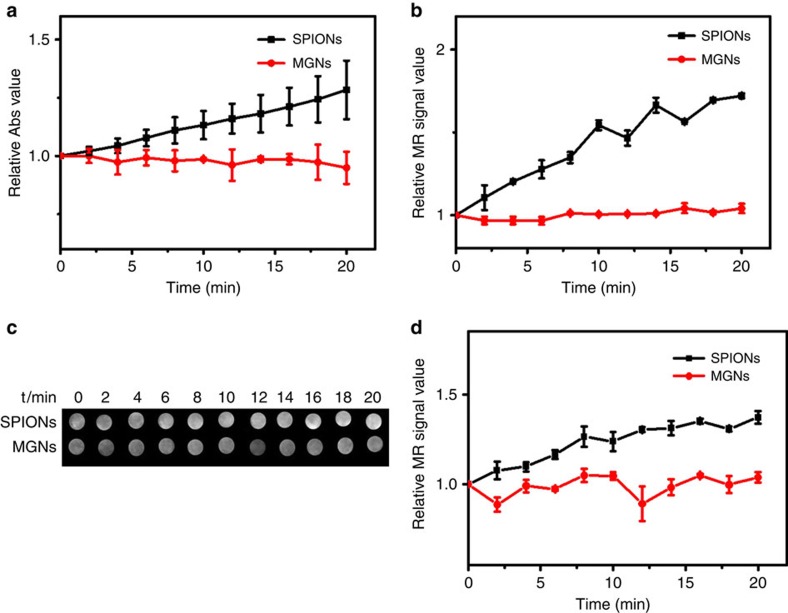# Corrigendum: *In situ* targeted MRI detection of *Helicobacter pylori* with stable magnetic graphitic nanocapsules

**DOI:** 10.1038/ncomms16154

**Published:** 2017-08-30

**Authors:** Yunjie Li, Xiaoxiao Hu, Ding Ding, Yuxiu Zou, Yiting Xu, Xuewei Wang, Yin Zhang, Long Chen, Zhuo Chen, Weihong Tan

Nature Communications 8: Article number: 15653; DOI: 10.1038/ncomms15653 (2017); Published: 06 15 2017; Updated: 08 30 2017

Supplementary Fig. 4 of this Article contains errors. Supplementary Fig. 4c shows the T_2_-weighted phantom images in reverse order, and Supplementary Fig. 4d is a duplicate of Fig. 3d. The corrected version of Supplementary Fig. 4 is shown below as Fig. 1.

## Figures and Tables

**Figure 1:**