# Supplementary material for: In situ targeted MRI detection of Helicobacter pylori with stable magnetic graphitic nanocapsules
Source: Nat Commun. 2017 Jun 15;8:15653. doi: 10.1038/ncomms15653 (PMC5501158; doi:10.1038/ncomms15653)
Supplement: Supplementary Information — Supplementary Figures and Supplementary Tables. [file ncomms15653-s1.pdf]

Supporting Information

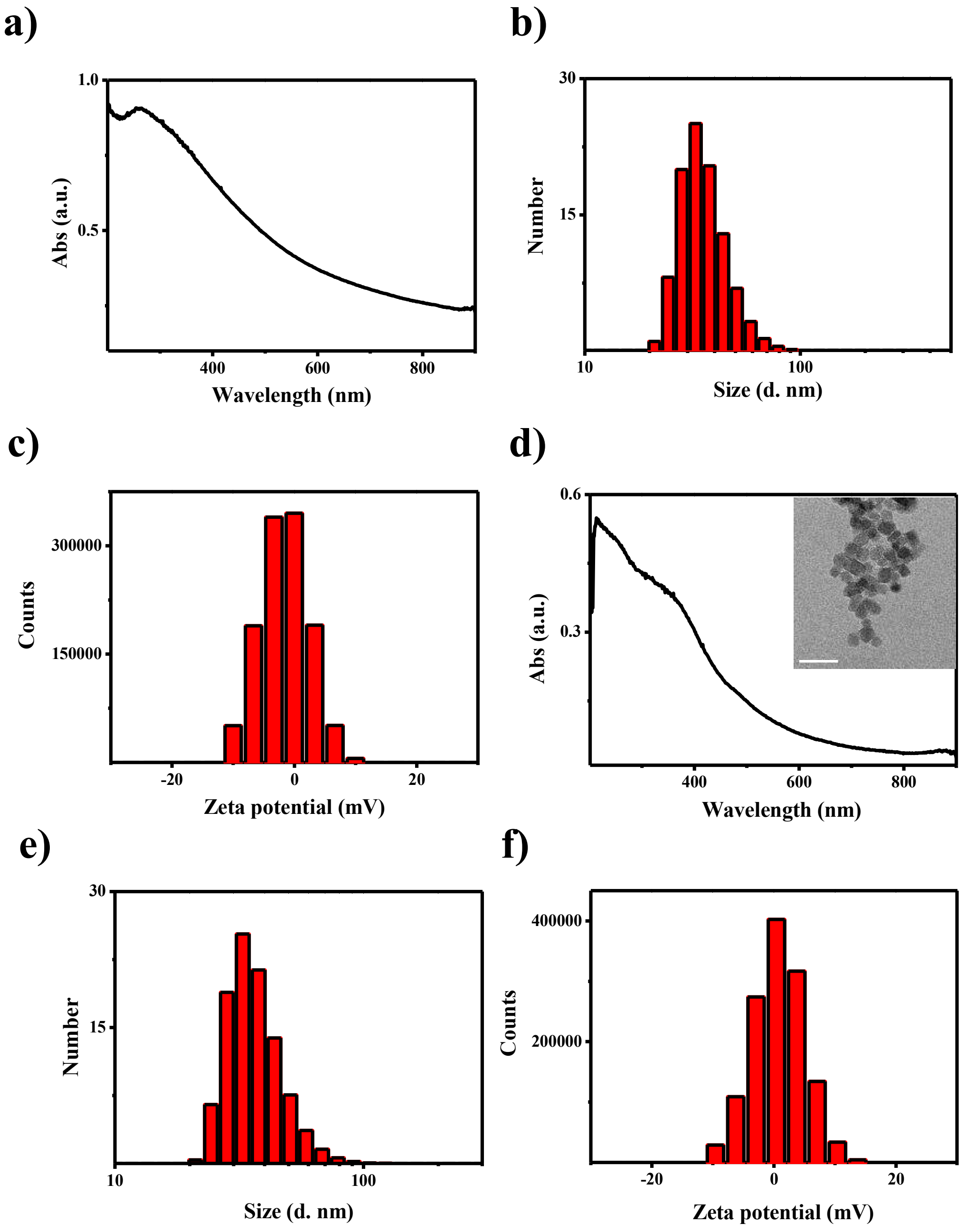

Supplementary Figure 1. Characterization of MGNs and SPIONs. (**a**) UV-Vis absorbance of MGNs. (**b**) Hydrodynamic diameter of MGNs characterized by dynamic light scattering. (**c**) Zeta potential of MGNs. (**d**) UV-Vis absorbance of SPIONs and TEM images of SPIONs as photo insert; scale bar, 20 nm. DLS (**e**) and zeta potential (**f**) characterization of SPIONs.

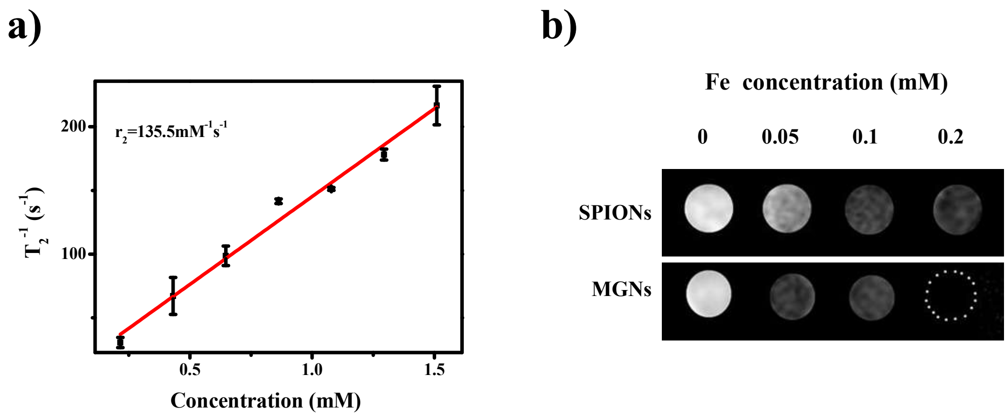

Supplementary Figure 2. Magnetic characterization. (**a**) MR T_2 measurement of SPIONs. The r_2 relaxivity value was obtained from the slope of the linear fit (red solid line) of the experimental T_2 data. (**b**) T_2-weighted phantom images of MGNs and SPIONs with different Fe concentrations.

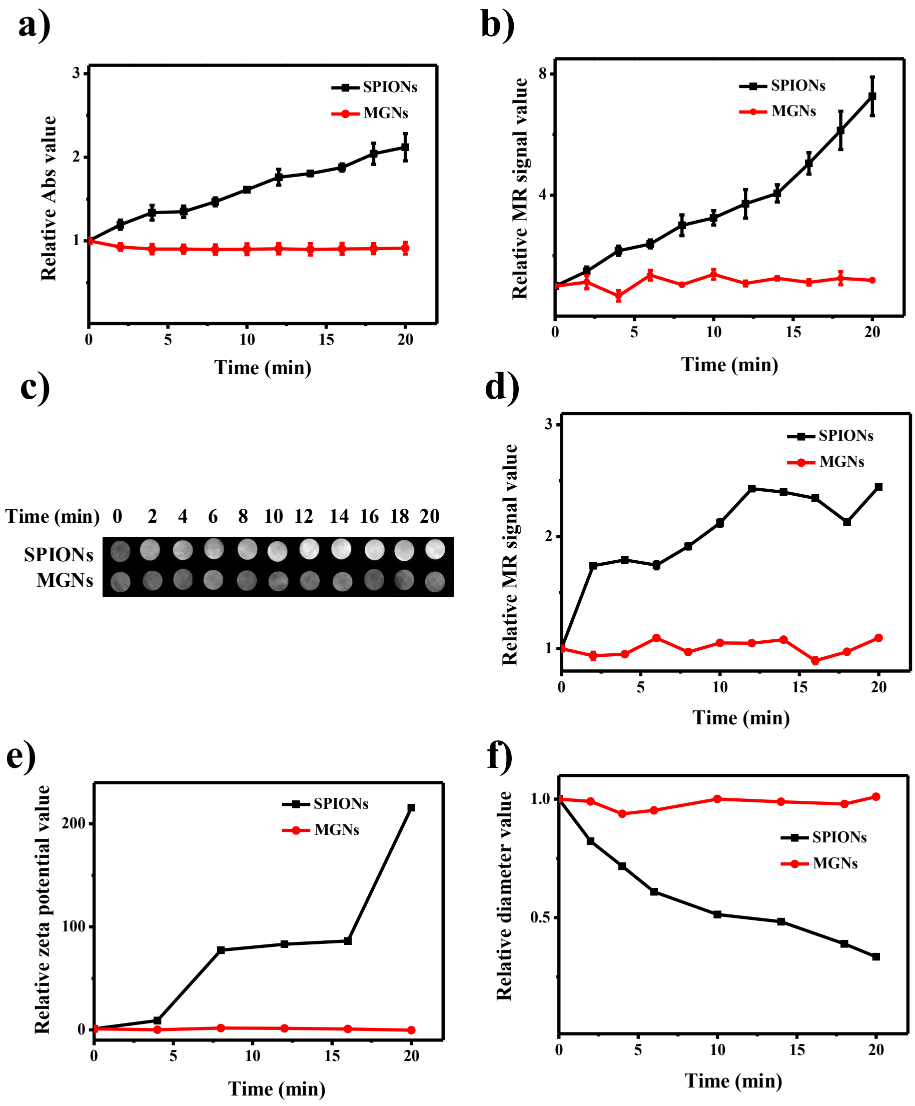

Supplementary Figure 3. Acid corrosion resistance tests with 4 M HCl. (**a**) UV-Vis absorbance at different time. (**b**) MR T_2 measurement at different time. (**c**) T_2-weighted phantom images at different time. (**d**) Corresponding data quantification analysis of (c) at different time. (**e**) and (**f**) DLS and zeta potential characterization of MGNs and SPIONs treated with 1 M HCl.

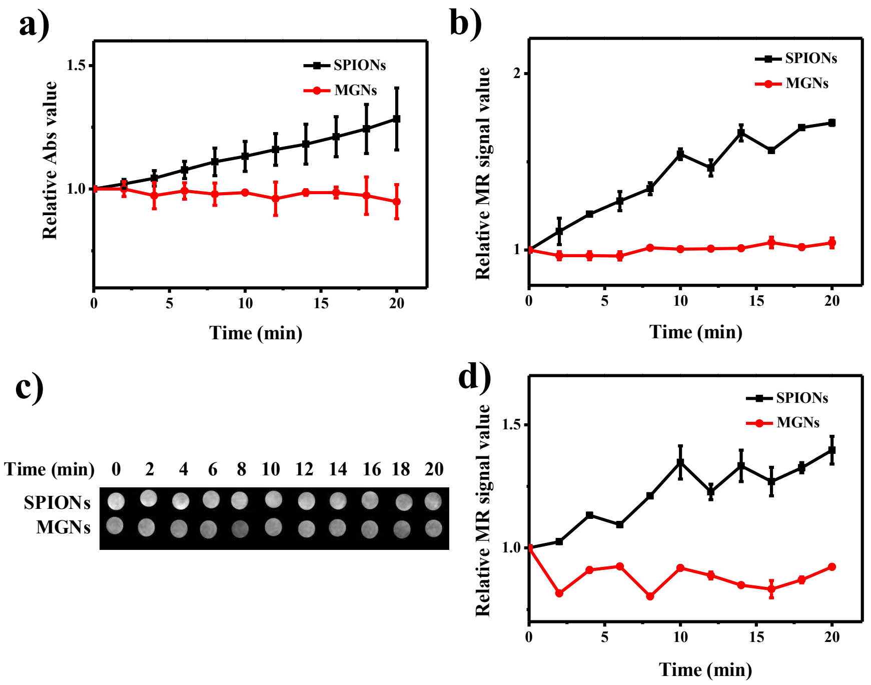

Supplementary Figure 4. Stability comparison of MGNs with pH = 1 gastric acid mimic. (**a**) and (**b**) UV-Vis absorbance and MR T_2 characterization. (**c**) T_2-weighted phantom images. (**d**) Corresponding data quantification analysis of (c) at different time after gastric acid treatments.

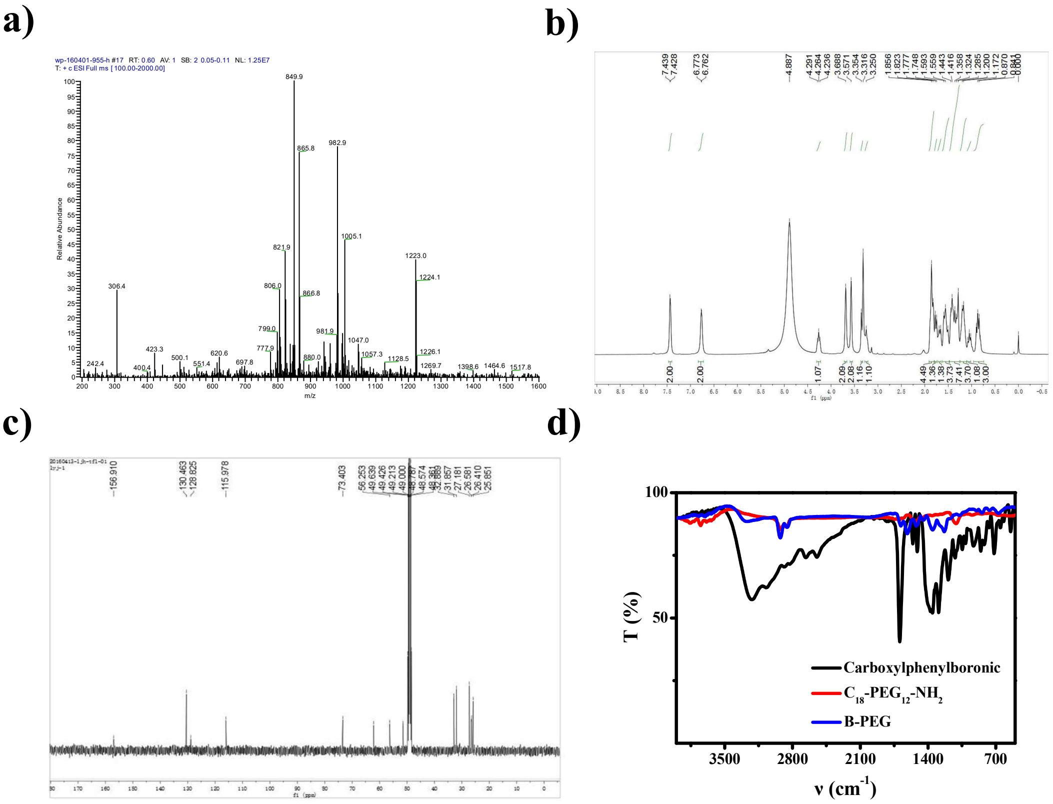

Supplementary Figure 5. Characterization of B-PEG. (**a**) ESI spectrum of B-PEG. (**b**) ^{1}H-NMR measurement with MQ 400 MHz. (**c**) ^{13}C-NMR measurement with MQ 400 MHz. (**d**) FTIR characterization of B-PEG.

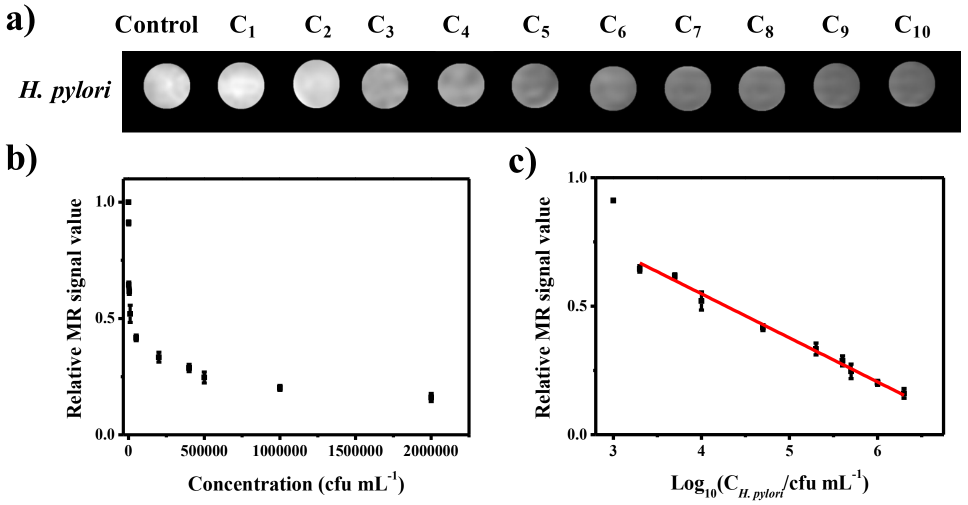

Supplementary Figure 6. Sensitive *H. pylori* detection *in vitro*. (a) MR imaging of different concentrations of *H. pylori* (C_1 to C_{10} are 1000, 2000, 5000, 1×10^4, 5×10^4, 2×10^5, 4×10^5, 5×10^5, 1×10^6, and 2×10^6 cfu mL^{-1}, respectively) incubated with MGN@B-PEG. **(b)** Corresponding relative MR signal values of (a). **(c)** The linear fit (red solid line) of relative MR signal value with the logarithmic *H. pylori* concentration.

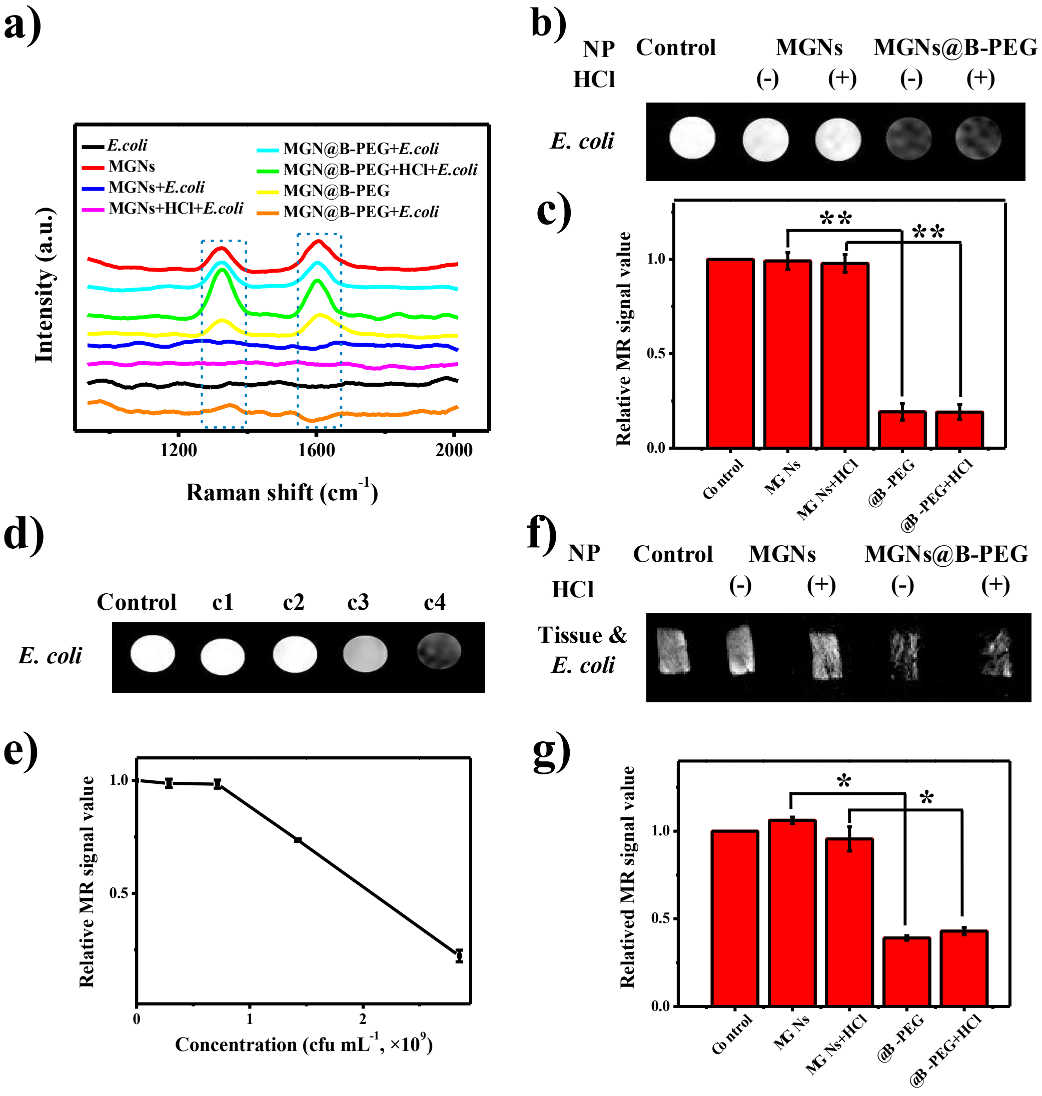

Supplementary Figure 7. Targeted *in vitro* detection of *E. coli* with MGN@B-PEG. (**a**) Raman spectroscopic characterization of *E. coli* detection with MGN@B-PEG. (**b**) MR images of *E. coli* with MGN or MGN@B-PEG treatments. (**c**) Corresponding quantitative data analysis of (b) with MGN or MGN@B-PEG treatments, (**P < 0.01 from the two-way ANOVA with Tukey's post-test). (**d**) MR images of different concentrations of *E. coli* with MGN@B-PEG. (**e**) Corresponding quantitative data analysis of (d) of different concentrations of *E. coli*. (**f**) MR images of *E. coli*-infected pork tissue with MGN or MGN@B-PEG after different treatments. (**g**) Corresponding quantitative data analysis of (f) with MGN or MGN@B-PEG after different treatments, (*P < 0.05 from the two-way ANOVA with Tukey's post-test).

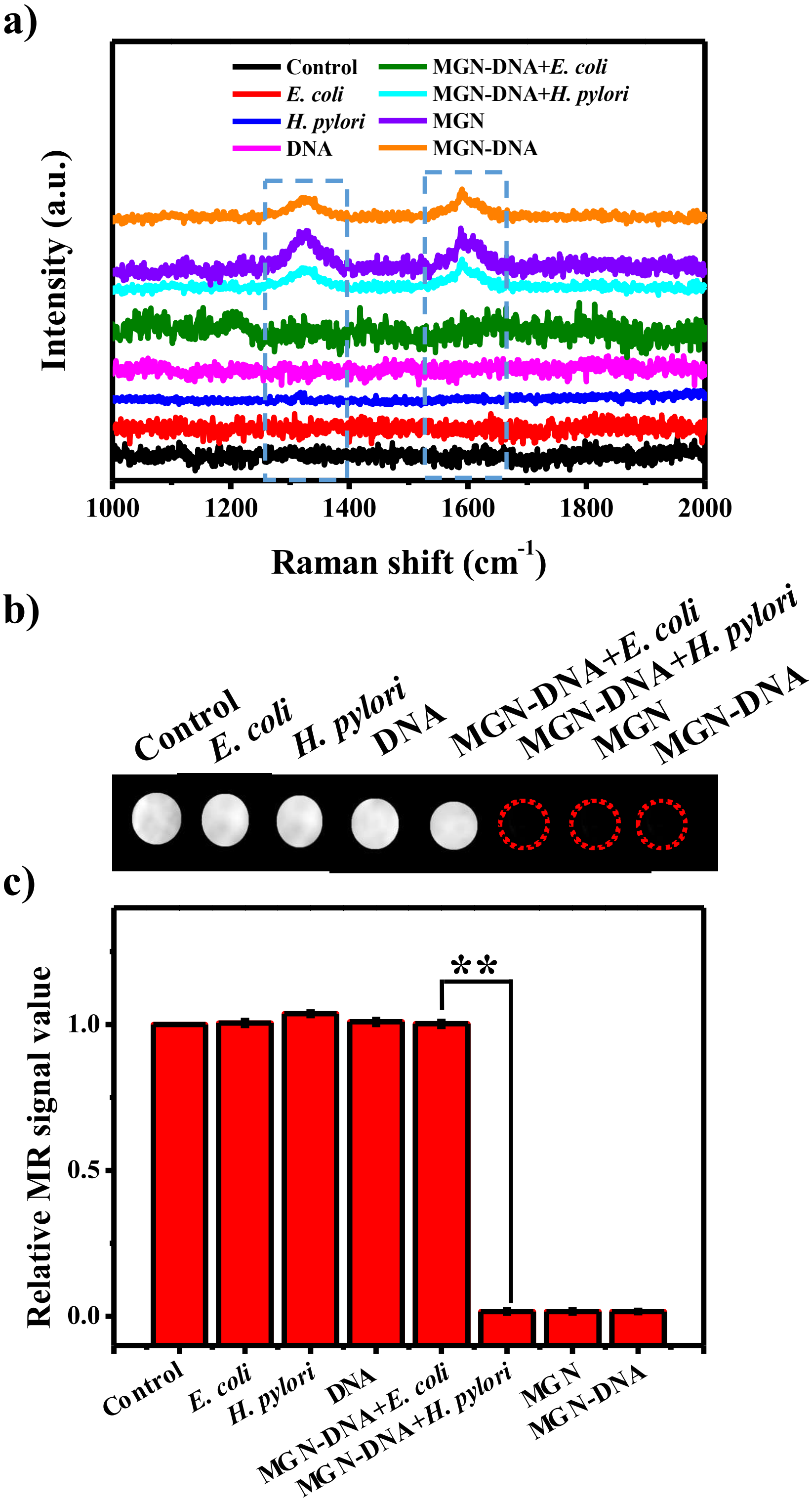

Supplementary Figure 8. **Nucleic acid functionalization for specificity of detection.** (**a**) Raman spectroscopic characterization of *E. coli* and *H. pylori* detection with MGN-DNA. (**b**) MR images of *E. coli* and *H. pylori* with MGN-DNA treatments. (**c**) Corresponding quantitative data analysis of (b) of *E. coli* and *H. pylori* with MGN-DNA treatments, (**$P < 0.01$ from the two-way ANOVA with Tukey's post-test).

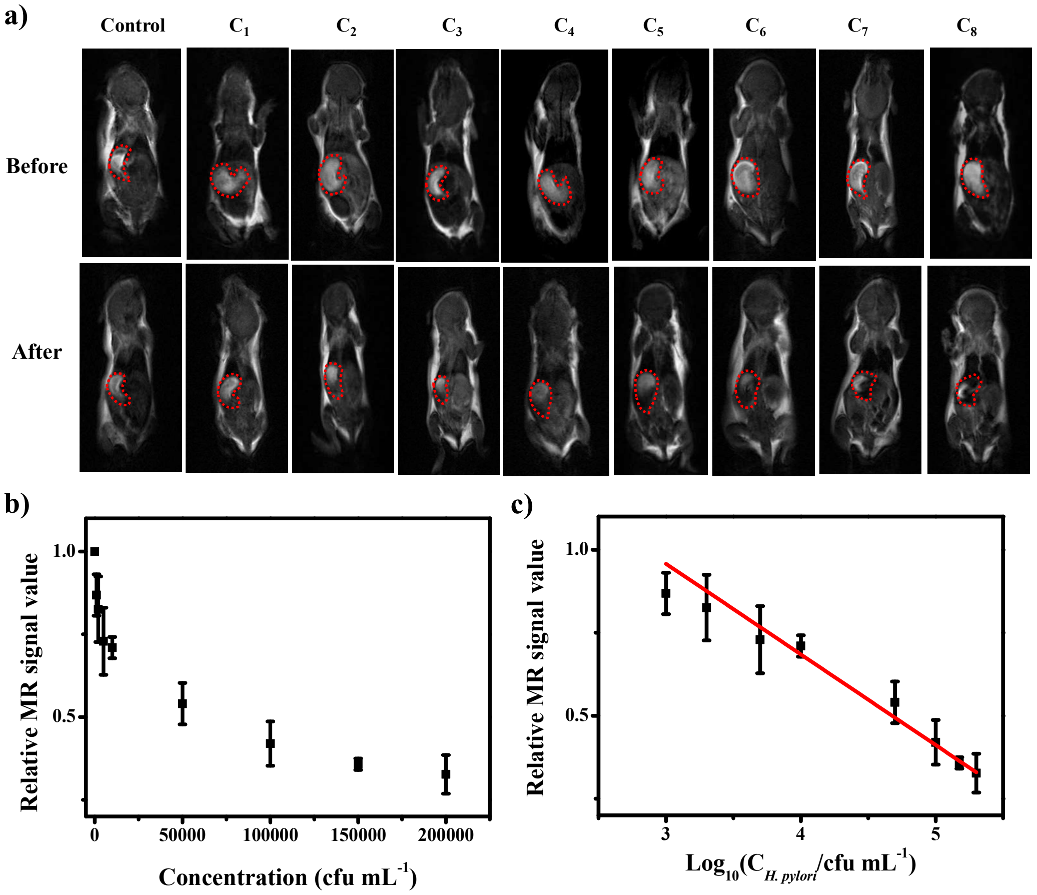

Supplementary Figure 9. Sensitive *H. pylori* detection in infected BALB/c mice. (**a**) T_2-weighted images of the mice with intragastric administration of different amounts of *H. pylori* (C_1 to C_8 are 1000, 2000, 5000, 1×10^4, 5×10^4, 1×10^5, 1.5×10^5, and 2×10^5 cfu mL^{-1}, respectively) before and 2 days after MGN@B-PEG treatment. (**b**) Corresponding data analysis of mice gastric mucosa of mice in (a) with intragastric administration of different amounts of *H. pylori*. (**c**) The linear fit (red solid line) of relative MR signal value with the logarithmic *H. pylori* concentration.

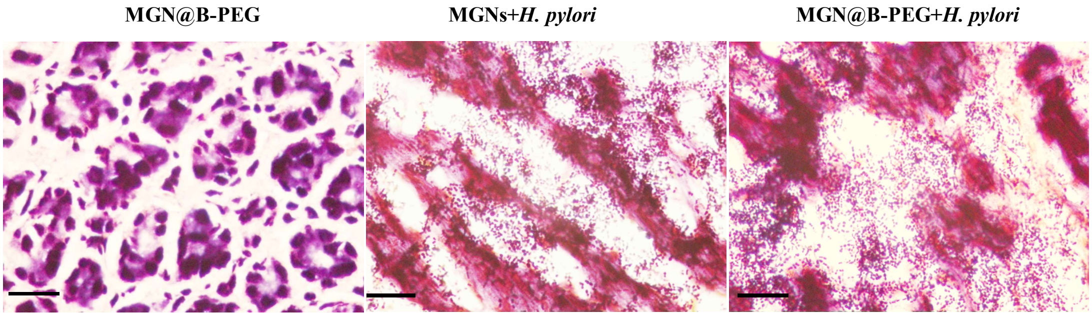

Supplementary Figure 10. **Gram staining of BALB/c mice gastric mucosa.** From the left to right: Gram-stained gastric slices of different mice corresponding to those in Figure 6b. Scale bar: 10 µm. Slice thickness: 10 µm.

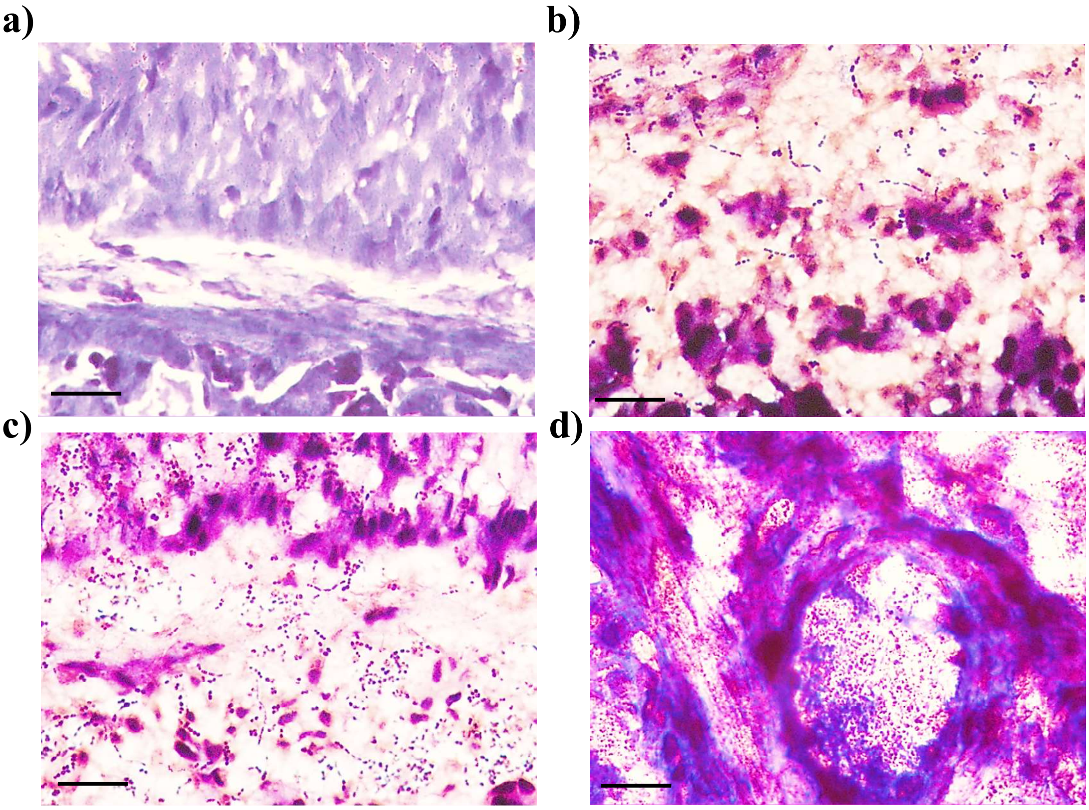

Supplementary Figure 11. **Gram staining of BALB/c mice gastric mucosa.** (**a**) Control: gastric mucosa of the mouse treated with DPBS. (**b**), (**c**) and (**d**) are the gastric mucosa of mice inoculated with 2000, 1×10^4 and 1×10^5 cfu mL^{-1} *H. pylori*, respectively. Scale bar: 10 μm. Slice thickness: 10 μm.

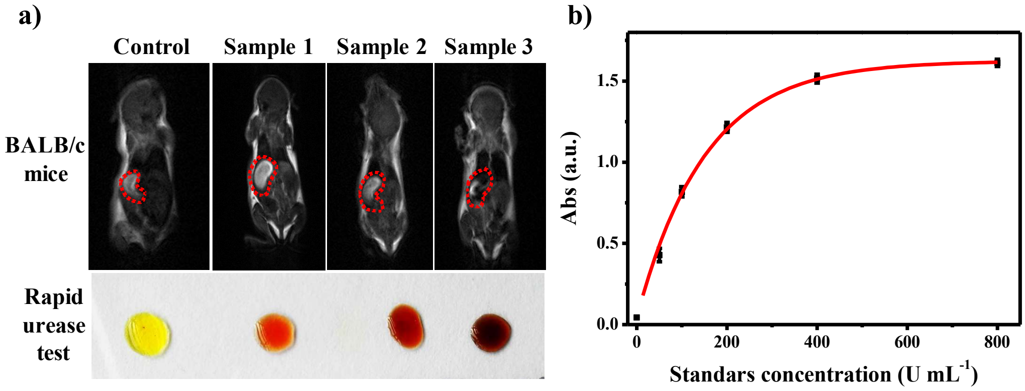

Supplementary Figure 12. Urease comparison tests. (**a**) Rapid urease tests of samples from mice with intragastric administration of *H. pylori*, (Control: 0 cfu mL^{-1}, Sample1: 2×10^5 cfu mL^{-1}, Sample2: 2000 cfu mL^{-1}+MGN@B-PEG, Sample3: 2×10^5 cfu mL^{-1}+MGN@B-PEG). The upper are the MRI images of the corresponding mice. (**b**) The standard curve from standard solutions with urease concentrations of 0, 50, 100, 200, 400, 800 U mL^{-1} (The equation of the standard solution is $y = -1.58\exp(-x/150.9)+1.62$, and the R-squared values is 0.9997).

	Control	Sample 1	Sample 2	Sample 3
Absorbance	0.04	0.9	0.5	1.25
Sample urease concentration	0 U mL^{-1}	900 U mL^{-1}	400 U mL^{-1}	1100 U mL^{-1}

Supplementary Table 1. Urease concentration calculated from the standard curve and corresponding absorbances at 450 nm of the samples in Figure 12a.
